# Novelty detection in long-term attentional habituation processes using a Bayesian change point algorithm

**DOI:** 10.1186/1471-2202-14-S1-P377

**Published:** 2013-07-08

**Authors:** Zeinab Mortezapouraghdam, Farah I Corona-Strauss, Tanja Teuber, Garbiele Steidl, Daniel J Strauss

**Affiliations:** 1 Department of Computer Science, University of Saarlandes, D-66123, Saarbrücken, Germany; 2 Saarland University, Faculty of Medicine, D-66421, Homburg/Saar, Germany; 3 Department of Mathematics, Technical University Kaiserslautern, D-67663 Kaiserslautern, Germany

## Introduction

Habituation is defined as a response decrement resulting from repeated stimulation [1]. This process is very important in filtering a large amount of information in the surrounding environment and focusing our attention on only the features of the environment that are important to us. Here we address the problem of objectively assessing long-term habituation using electroencephalographic auditory late responses (ALRs). We propose using a Bayesian change point algorithm to measure the novelty process and apply it to two experimental data sets of 50dB and 100dB stimuli responses. These correspond to cases of habituation and non-habituation respectively.

## Methods

We use unprocessed raw data along with a denoised variant of the same data for the analysis. The nonlocal means algorithm is used for 2 diemensional denoising of ALR single sweeps, arranged in matrix, see [2] for detials. Data consists of 900 artefact free sweeps of 410 samples. We analyzed the data at all time instances over all sweeps. This procedure is applied to the unprocessed and processed data set. The Bayesian change point detection algorithm estimates a probability distribution over a run-length parameter. The run-length parameter has two states. It either increases by one time-step, or resets to zero indicating a change point. We calculate an average run-length variation value for all signals, based on changes in the maximum likelihood run-length. This provides a metric for comparing habituation and non-habituation processes.

## Conclusion

**Figure 1:**
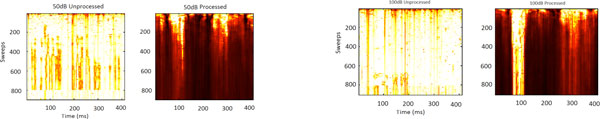
**The following images show the likelihood of change points over all ALR sweeps arranged in matrix**. The run-length likelihood is computed recursively by$p\left(r_{t},a_{t}|x_{1:t}\right)=p\left(r_{t},a_{t},x_{1:t}\right){\left(\sum_{r_{t}}\sum_{a_{t}}p\left(r_{t},a_{t},x_{1:t}\right)\right)}^{-1}$. The resulting likelihoods for having change point are plotted, (brighter corresponds to higher likelihoods). There is more variations in the likelihood of change points in 50dB throughout 80-120 ms across the sweeps. For 100dB the likelihood of change point remains the same through all sweeps indicating no habituation is occurring.

The results illustrate that the preprocessing method can significantly enhance the results of the Bayesian change-point algorithm. The algorithm is likely to fail in case of the unprocessed version of the data. After noise-suppression, the behavior of run-length variations over sweeps is expressed more clearly. Also, on average (9 out of 11 data set) there exists higher change point variations for 50dB compared to 100dB responses. This is illustrated in Figure 1 where the likelihood of change points tends to remain at a high value through all sweeps indicating a constant state generation. The finding illustrates that there exists a higher level of novelty degree involved in case of habituation (50dB) compared to non-habituation (100dB).
